# Early, On-Treatment Levels and Dynamic Changes of Genomic Instability in Circulating Tumor DNA Predict Response to Treatment and Outcome in Metastatic Breast Cancer Patients

**DOI:** 10.3390/cancers13061331

**Published:** 2021-03-16

**Authors:** Adriana Aguilar-Mahecha, Josiane Lafleur, Susie Brousse, Olga Savichtcheva, Kimberly A. Holden, Nathan Faulkner, Graham McLennan, Taylor J. Jensen, Mark Basik

**Affiliations:** 1 Lady Davis Institute, Jewish General Hospital, Montreal, QC H3T 1E2, Canada; adriana.aguilar@mail.mcgill.ca (A.A.-M.); jlafleur@jgh.mcgill.ca (J.L.); susie.brousse@gmail.com (S.B.); osavichtcheva@gmail.com (O.S.); 2 Laboratory Corporation of America, Burlington, NC 27216-2240, USA; Kellk10@labcorp.com (K.A.H.); Faulkn3@labcorp.com (N.F.); Mclenng@labcorp.com (G.M.); Jenset2@labcorp.com (T.J.J.)

**Keywords:** ctDNA, metastasis, whole-genome sequencing, plasma, breast cancer, genomic instability, clinical response

## Abstract

**Simple Summary:**

Liquid biopsies offer the opportunity to monitor cancer progression and the response to treatment with a simple blood test. However, most of the technologies available analyze specific molecular alterations or require tumor tissue for analysis, which is very difficult to obtain in metastatic patients. In this study, we made use of a novel method that allows to measure the overall molecular tumor changes in a blood sample without the need for tissue or to look for specific molecular alterations. We demonstrated the ability of this method to very early monitor the treatment clinical response and progression in a cohort of metastatic breast cancer patients.

**Abstract:**

Background: Circulating tumor DNA (ctDNA) offers high sensitivity and specificity in metastatic cancer. However, many ctDNA assays rely on specific mutations in recurrent genes or require the sequencing of tumor tissue, difficult to do in a metastatic disease. The purpose of this study was to define the predictive and prognostic values of the whole-genome sequencing (WGS) of ctDNA in metastatic breast cancer (MBC). Methods: Plasma from 25 patients with MBC were taken at the baseline, prior to treatment (T0), one week (T1) and two weeks (T2) after treatment initiation and subjected to low-pass WGS. DNA copy number changes were used to calculate a Genomic Instability Number (GIN). A minimum predefined GIN value of 170 indicated detectable ctDNA. GIN values were correlated with the treatment response at three and six months by Response Evaluation Criteria in Solid Tumours assessed by imaging (RECIST) criteria and with overall survival (OS). Results: GIN values were detectable (>170) in 64% of patients at the baseline and were significantly prognostic (41 vs. 18 months OS for nondetectable vs. detectable GIN). Detectable GIN values at T1 and T2 were significantly associated with poor OS. Declines in GIN at T1 and T2 of > 50% compared to the baseline were associated with three-month response and, in the case of T1, with OS. On the other hand, a rise in GIN at T2 was associated with a poor response at three months. Conclusions: Very early measurements using WGS of cell-free DNA (cfDNA) from the plasma of MBC patients provided a tumor biopsy-free approach to ctDNA measurement that was both predictive of the early tumor response at three months and prognostic.

## 1. Introduction

Metastatic breast cancer is mostly incurable, and patients are treated with serial chemotherapy regimens to prolong life and decrease the symptoms. The median survival is usually in the range of two years, and oncologists strive to limit the toxicity by stopping therapies when the response is judged inadequate and switching to either the next-line treatment or initiating palliative care. The prognosis of patients with metastatic breast cancer is dependent on the response to therapy, as well as other factors, including the number of metastatic sites, the location of metastases (bone only vs. extra-osseous), the patient’s age, the clinical presentation and the breast cancer subtype [1,2,3,4]. Indeed, the recent development of novel anti-HER2-targeted agents looks to significantly improve the prognosis of patients with this breast cancer subtype [5,6]. The treatment efficacy is usually monitored by imaging every three months—at which time, decisions are made about continuing therapy. Novel approaches to monitoring the treatment response and estimating the prognosis are needed to enable timely treatment decisions.

The use of liquid biopsies is a novel diagnostic method that has high sensitivity and specificity in metastatic cancer [7]. For instance, the detection of circulating tumor DNA (ctDNA) in plasma can anticipate the clinical tumor progression by a median lead time of 8–11 months [8,9]. Assays based on the detection of tumor-specific mutations in plasma have led to the FDA approval of companion diagnostic tests linked to targeted therapies [7,10]. These assays are, in large part, based on the detection of either known recurrently mutated genes or single mutational hotspots in these genes. The detection of such common variants provides an alternative to sequencing of the tumor tissue in contexts in which the prevalence of these variants is frequent, such as the detection of KRAS mutations in colorectal cancers [11] and Epidermal Growth Factor Receptor (EGFR)mutations in lung cancer [12]. The detection of private mutations or infrequently mutated genes is more difficult to apply in the clinic because of the necessity of first sequencing the tumor [13]. Moreover, the primary tumor may have evolved during the metastatic process, and accessing metastatic tumors is difficult [14,15].

One way to solve this problem is to use a large panel of commonly mutated genes or a genome-wide approach. The Genomic Instability Number (GIN) is a recently published measure of DNA copy number changes across the genome using low-coverage, genome-wide sequencing of cell-free DNA (cfDNA) [16]. As it is genome-wide, it does not depend on tumor sequencing. In this study, we tested the GIN in plasma from a cohort of metastatic breast cancer patients undergoing chemotherapy and correlated the GIN levels with the clinical response and outcome data. Our results showed that the baseline, early, on-treatment GIN values and dynamic changes can predict the response to treatment, as well as the overall survival. 

## 2. Materials and Methods

### 2.1. Patients

Metastatic breast cancer patients (*n* = 39) were recruited between 2016 and 2017 at the Jewish General Hospital (JGH) in Montréal, QC, Canada. Patients presenting any type of breast cancer and undergoing any line of chemotherapy were eligible. All participants provided informed consent, and the study was approved by the JGH Ethics Committee Review Board and complied with the local ethics guidelines (Protocol#15-121, 21-09-2015). Patients consented to be part of a serial plasma collection protocol at different follow-up time points. Clinical response to treatment at 3 and 6 months after treatment initiation was determined from physician assessment (e.g., for skin lesions) and radiographic review when available. We used Response Evaluation Criteria in Solid Tumours assessed by imaging (RECIST) criteria to classify patients with complete response (CR), partial response (PR) or stable disease (SD) as responders and patients with progressive disease (PD) as non-responders. 

### 2.2. Sample Collection and Processing

At each time point, blood was collected in two 10-mL Cell-Free DNA BCT tubes (Streck, Montreal, QC, Canada) during medical appointments at the JGH in Montreal, QC, Canada and then shipped to the central lab (Sequenom, San Diego, CA, USA) for separation of the whole blood into plasma using centrifugation, as previously described [16]. 

Matching frozen tumor tissue was available for a subset of patients through the JGH breast biobank (protocol 05-006). Tumor cellularity was verified by a pathologist and confirmed to be >50%. DNA extraction from frozen tumor tissue was performed using Qiagen Allprep DNA/RNA kit (Qiagen, Germantown, MD, USA). 

### 2.3. cfDNA Extraction and Quantification

cfDNA from the plasma of each sample (~4 mL) was extracted using a bead-based method, as previously described [17]. 

### 2.4. Sequencing Library Preparation

Libraries for genome-wide sequencing were created from cfDNA, as previously described [15]. Libraries from genomic DNA obtained from frozen tumor tissue were created using the same process; however, DNA was randomly fragmented using ultrasonication prior to the initiation of library preparation.

### 2.5. Genome-Wide Next-Generation Sequencing

Normalized sequencing libraries were pooled and sequenced using HiSeq2500 (Illumina, San Diego, CA 92122, USA) instruments, as previously described [16]. A mean of 32.8 million sequencing reads (~0.3× genomic coverage) were generated for each sample.

### 2.6. Sequencing Data Analysis

Sequencing data were processed to calculate a genomic instability number (GIN) based on the presence of copy number alterations (CNAs), as previously described [16]. Briefly, the GIN is a measure that represents the cumulative deviations of all copy number alterations across the genome. Hence, it is influenced by both the magnitude of the CNAs present in the tumor and the level of ctDNA in the plasma. Previous work on plasma samples from 6014 noncancer patients undergoing neonatal DNA testing was used to determine a threshold for GIN detection of 170 [16]. 

### 2.7. Statistical Analysis

Correlation of GIN scores with clinical outcome was performed at the Basik Laboratory (JGH, Montreal, QC, Canada). The performing laboratory was blinded to patient outcomes until after the analysis was complete. The GIN value at baseline (before the start of chemotherapy), as well as its variation 1 and 2 weeks after the start of therapy, were compared with the treatment response at 3 and 6 months, PFS and OS. The *p*-value for quantitative GIN comparison according to the different characteristics or outcomes was calculated using a two-tailed Student’s *t*-test to compare means or a Kruskal–Wallis test to compare medians. Kaplan–Meier curves were constructed for PFS and OS, and log-rank tests were performed to determine the equality of survival distributions. All reported *p*-values were two-sided and considered statistically significant when <0.05. Statistical analyses were performed with Graph Pad Prism (Version 6, Graph Pad Software, San Diego, CA, USA). Sensitivity, specificity, positive predictive value (PPV), negative predictive value (NPV) and 95% confidence interval (CI) were calculated using the MEDCALC online tool (https://www.medcalc.org/calc/diagnostic_test.php, accessed on 18 November 2020)

## 3. Results

### 3.1. Patient Population

Patients with metastatic breast cancer initiating a new treatment line consented to provide blood prior to the beginning of a new line of therapy (T0), at T1, an average of one week (5–9 days), and at T2, an average of two weeks (11–21 days), post the treatment initiation. Of the 39 recruited patients, 14 were excluded because of no baseline and/or serial blood samples, withdrawal of consent or cessation of treatment before the first clinical assessment (three months) (Figure S1). In total, two to three serial blood samples from 25 metastatic breast cancer patients were included in this analysis (Figure S1). Breast cancers of all three subtypes were represented, nine hormone receptor-expressing (HR+), nine triple-negative breast cancer (TNBC) and seven HER2 amplified or overexpressed (HER2+) (Table S1). All but one had measurable or observable metastatic diseases. That patient had resection of the brain metastases and radiotherapy of the bone metastases prior to the initiation of HER2-directed therapy, without evidence of disease at all at clinical follow-ups up to five years, and was considered to have a complete response.

The treatment response was assessed at three and at six months, and we grouped patients with a response and stable disease as the responder group (R) vs. PD patients for further analyses. Two patients who died before three months and three patients who died before six months were counted as PD. A total of five patients and 13 patients had PD at three months and six months, respectively. The median progression-free survival and overall survival were seven months (range = 0–42) and 24 months (range = 1–46), respectively (Table S1). 

### 3.2. GIN Is Measurable in the Plasma of Metastatic Breast Cancer Patients

**Figure 1:**
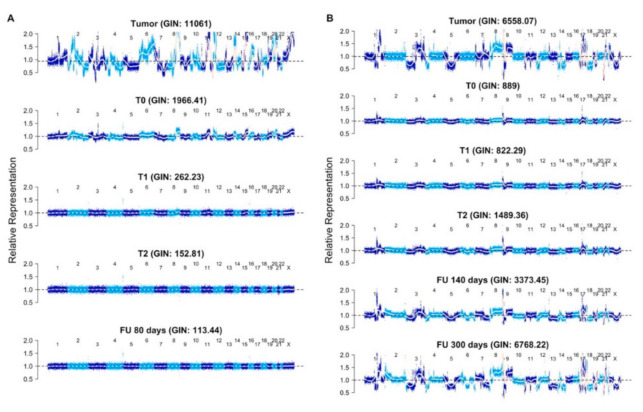
Genome-wide representation of the profile obtained from low-pass whole-genome sequencing of the tumor DNA (top sample) and serial plasma samples (T0 (prior to treatment), one week (T1), two weeks (T2)—as defined in the text—and a follow-up (FU)) plasma sample obtained 80, 140 or 300 days after treatment initiation. The Genomic Instability Index (GIN) is indicated above each sample. The profiles show the Log2 DNA copy number values relative to the diploid 1.0 value for each chromosome from 1 to X, left to right. (**A**) Patient showing a response to treatment. (**B**) Patient showing a progressive disease. Note that the profile in the plasma at 300 days is very similar to the tumor’s DNA copy number profile in the nonresponding patient, whereas the plasma genomic profile progressively flattens out after treatment initiation in the responding patient.

Based on the predetermined threshold of GIN > 170 as being elevated about the baseline and, thus, determined to be “detectable”, we found a detectable GIN in at least one sample from 17 of 25 metastatic patients (68%). Examples of the GIN results for two patients are shown in Figure 1. In both of these cases, the same genomic analysis was performed on a matched tumor, showing very good concordance between the tumor and the plasma profiles, including the detection of Human Epidermal Growth Factor Receptor 2 (ERBB2) amplification in the samples from patient 15 (Figure 1B). 

To evaluate the analytical limit of detection using the established GIN = 170 threshold, dilutions of the three different patient tumor DNA samples in normal healthy plasma DNA were prepared. Since the number and/or magnitude of copy number alterations in a tumor genome may vary, the exact proportion of tumor DNA necessary to be present in the cfDNA to yield a GIN value of greater than 170 may be patient-dependent. The Loess regression analysis was performed on the data to determine the proportion of tumor DNA needed to meet or exceed the GIN = 170 threshold for patients 3, 15 and 21. The proportion required to observe a GIN ≥ 170 for patient 3 was 0.93%, patient 15 was 1.68% and patient 21 was 1.26%; this allowed us to determine that as little as 1% to 2% of tumor DNA is sufficient to reach a detectable GIN value in cfDNA (Figure S2). The one patient with no measurable/observable disease before treatment did not have a detectable GIN at any time point, consistent with the clinical outcome for this patient (Figure S3).

### 3.3. GIN Scores at Baseline Are Associated with Survival

**Figure 2:**
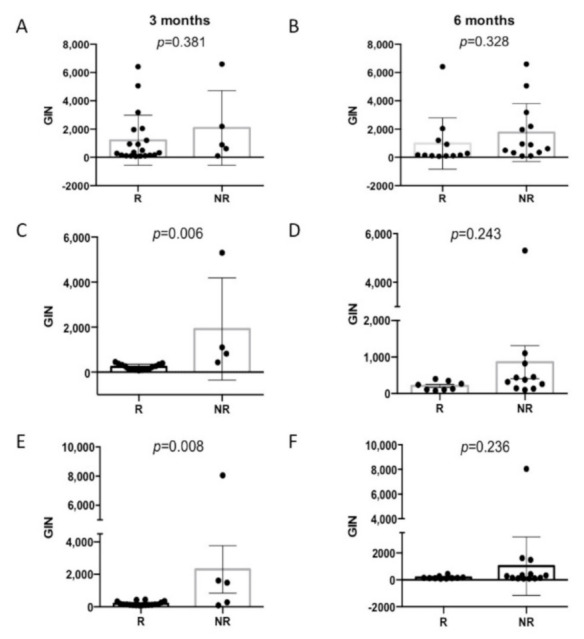
GIN values at T1 and T2 are associated with the 3-month clinical response. Scatter box plots of GIN values in responding patients (R) compared with nonresponding patients (NR). Clinical Response at 3 months: (**A**) (T0 or baseline), (**C**) (T1 values) and (**E**) (T2 values). Clinical response at 6 months: (**B**) (T0 or baseline), (**D**) (T1 values) and (**F**) (T2 values). The error bars reflect the standard deviations.

The plasma GIN values at the pretreatment baseline (T0) ranged from 80 to 6599 (Median = 492). The GIN was detectable (>170) at the baseline in 16 out of 25 patients (64%). The baseline plasma GIN levels were not associated with the clinical features, including the number of metastatic sites and the number of lines of treatment (Figure S4). The average baseline GIN level was not associated with the treatment response at three months (*p* = 0.38) or at six months (*p* = 0.33) (Figure 2A,B). 

**Figure 3:**
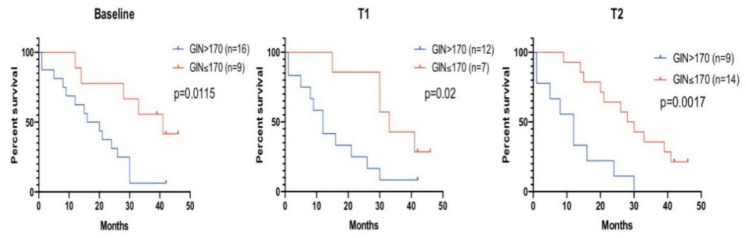
Overall survival and GIN detection at different time points. The overall survival of patients with plasma GIN values above and below the pre-established threshold (170) at 3 different time points. Left: Baseline, middle: T1 (average 1 week post-treatment) and right: T2 (average 2 weeks post-treatment).

We evaluated the PFS and OS according to GIN detectability at the baseline. Although GIN detection was not significantly associated with PFS (Figure S5), patients with detectable baseline GIN had a worse overall survival (median = 18 months) than patients without a detectable GIN at baseline (median = 41 months) (*p* = 0.016) (Figure 3). Interestingly, in eight out of nine patients with GIN values below the detection level at baseline, the GIN remained undetectable at all subsequent time points where plasma was available. Only one patient with a nondetectable GIN value at the baseline showed an increase in GIN over the established threshold at T1 and T2. This patient showed a stable disease at three and six months. 

### 3.4. Early On-Treatment GIN Scores Are Associated with Treatment Response and Outcome

To obtain data on the very early treatment-related changes in GIN, we collected blood an average of one and two weeks after treatment initiation. Since it was difficult to schedule blood draws on exactly the same day post-treatment, we divided the blood draw time points into two time intervals: within 10 days (T1) and after day 10 but within 22 days after treatment initiation (T2). The median timing for the first group was seven days (range five to nine days) and the second group, 14 days (range 11–21). A total of 19 patients had T1 blood samples, while 23 patients had T2 blood samples. Seventeen patients had samples analyzed from all the evaluated time intervals, including the baseline. The GIN values at T1 ranged from 78–5300 (median = 267) and at T2 from 94–8050 (median = 149), with 12/19 patients with detectable GIN at T1 and 10/23 patients with detectable GIN at T2.

We found a significant association between GIN values at T1 and T2 with a three-month clinical response. The average GIN value at T1 was 231 for three-month responders vs. 1916 for patients with PD at three months (*p* = 0.006) (Figure 2C). Similarly, the average GIN value at T2 was 196 in three-month responders, significantly lower (*p* = 0.008) than the average GIN value at T2 (2308) in patients with PD at three months (Figure 2E). In contrast, the average GIN values at T1 and T2 were not associated with the six-month response (Figure 2D,F). 

The presence of detectable GIN values (>170) at T1 and T2 was associated with a significantly lower OS. Patients with detectable GIN at T1 had a median survival of 12 vs. 33 months for patients with undetectable GIN (Hazard Ratio (HR), 3.01; *p* = 0.009). At T2, the median survival was 12 months for GIN > 170 and 29 months for patients with undetectable GIN (HR, 3.48; *p* = 0.001) (Figure 3). No association between the PFS and detectable GIN at T1 and T2 was found (Figure S5).

### 3.5. Early GIN Score Changes during Treatment 

#### 3.5.1. Baseline vs. T1

**Figure 4:**
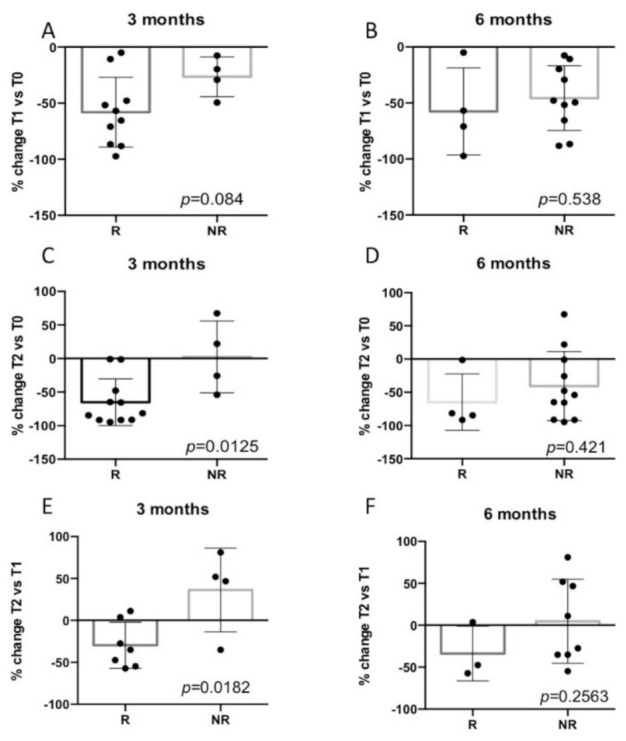
Percent (%) change in GIN values between T2 and T0 and T2 and TA are associated with a 3-month clinical response. Scatter box plots of the % change in GIN in responding patients (R) compared with nonresponding patients (NR). Clinical response at 3 months: (**A**) T1 vs. T0, (**C**) T2 vs. T0 and (**E**) T2 vs. T1. Clinical response at 6 months: (**B**) T1 vs. T0, (**D**) T2 vs. T0 and (**F**) T2 vs. T1. The error bars reflect the standard deviations.

For 14 patients with a detectable GIN at the baseline and blood available at T1, the average decrease in GIN was 49%, with three of these reaching nondetectable levels. We assigned a GIN score of 170 to all nondetectable GIN values and found that three-month responders with a detectable baseline GIN had a mean 58% decrease in their GIN at T1, while PD patients only had a 26% decrease (Figure 4A) (*p* = 0.08). Looking at the six-month response, there was no significant difference in the average % GIN change at T1 for responders vs. PD (−58% vs. −46%, *p* = 0.53, Figure 4B).

All seven patients with a GIN decline at T1 of ≥50% were three-month responders (Table S1), but four of these went on to PD at six months. On the other hand, six out of seven patients with smaller declines in GIN at T1 (<50%) had PD at six months.

**Figure 5:**
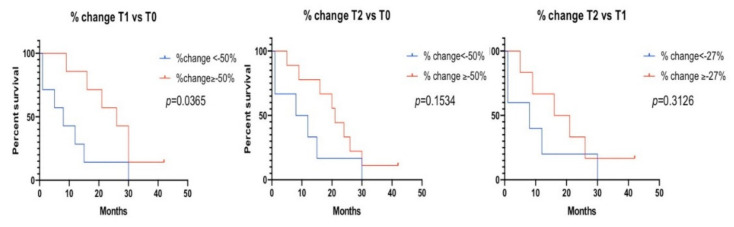
Overall survival and percent (%) change in GIN values at different time points. The overall survival of patients with a plasma % change in GIN values above and below the pre-established threshold (−50%) or the median (−27%). Left: % change T1 vs. T0, middle: T2 vs. T0 and right: T2 vs. T1.

An association between a decreased GIN at T1 and overall survival was identified. The median overall survival for patients with GIN detected at the baseline and decreased at T1 below a set threshold of −50% was eight months vs. 26 months for those with a decrease at T1 greater than −50% (HR, 2.70; 95%CI, 1.27 to 13.54; *p* = 0.037) (Figure 5). No relationship between a decline in GIN at T1 and PFS was found (Figure S5). 

#### 3.5.2. Baseline vs. T2

For 15 patients with a detectable GIN at the baseline and blood available at T2, the average decrease in GIN was 47%, with six dropping to nondetectable levels (Table S1). There was a significant difference in the mean % change in GIN values at T2 in three-month responders with initially detectable GIN compared to PD patients (−65% vs. +2%), *(p* = 0.012) (Figure 4C). Eight out of nine patients with a ≥50% decrease in GIN levels at T2 relative to the baseline showed a response at three months (Table S1). Although the % GIN changes from T2 to the baseline were not significantly different in responders vs. PD patients (Figure 4D), five out of six patients with smaller declines in GIN at T2 (<50%) were nonresponders. No association between the change in T0 vs. T2 was found with the overall survival (Figure 5) or PFS (Figure S5).

#### 3.5.3. T2 vs. T1 and GIN Patterns

The difference in GIN between one and two weeks on the treatment was also evaluated. If either the T1 or T2 GIN was below the detection threshold, we assigned a value of 170 to the ND value to calculate the difference. When both the T1 and T2 GIN values were not detectable, they were not included in the analysis (Table S1). The average decrease in GIN at T2 vs. T1 for patients with detectable GIN at the baseline was 6% (SD = 48). The % change between the two on-treatment time points was significantly different between the three-month responders (−36%) and nonresponders (+30%) (*p* = 0.0182) (Figure 4E). No association between the change in T1 vs. T2 was found with the response at six months (Figure 4F) or with the overall survival (Figure 5) or PFS (Figure S5).

By looking at the dynamic changes in the GIN for the 13 patients with detectable GIN at the baseline and GIN measured at T1 and T2, two primary patterns were discerned. The first was characterized by a decrease in the GIN level at T1, followed by either a decrease or no change at T2, and the second by a decrease in GIN at T1, followed by an increased GIN at T2 (V-shaped). Seven out of eight patients with the first pattern had a clinical response at three months, while three out of five patients with the second pattern had PD at three months and four at six months. (Figures S6 and S7). 

### 3.6. GIN Is Higher and Decreases More Dramatically in TNBC Patients

We looked at the GIN values separately in the three breast cancer (BC) subtypes (hormone receptor-expressing (HR+) (*n* = 9), HER2 amplified or overexpressed (HER2+) (*n* = 7) and triple-negative breast cancer (TNBC) (*n* = 9). The TNBC patients had a significantly higher median baseline GIN than those with HR+ or HER2+ cancers (1966 vs. 357 vs. 156) (*p* = 0.025, Figure S8). Only two HER2+ BCs had detectable GIN values compared to six HR+ BCs and eight TNBC patients.

For patients with a detectable GIN at the baseline, the absolute magnitude of the mean change in GIN at T1 was greater in TNBC patients (−71%) compared to HR+ BCs (−32%) (*p* = 0.014) (Figure S8). At T2, the change in GIN was −26% in HR+/HER2+ BCs and −76% in TNBCs (*p* = 0.027). Thus, although patients with TNBC appear to have higher GINs before treatment, the treatment results in a more pronounced decrease in GIN values in TNBCs.

## 4. Discussion

The early detection of a therapeutic response holds the promise of enabling timely and effective treatment decisions that may minimize the cost and toxicity of ineffective treatments and maximize the time on effective therapy. Presently, decisions about treatment efficacy are made by oncologists on the basis of radiological tumor imaging, typically performed three months after treatment initiation. The measurement of circulating tumor DNA (ctDNA) enables a highly sensitive and specific quantification of tumor burden in advanced solid tumors [18,19,20]. Recent reports have suggested that early (e.g., 8–21 days after treatment initiation) declines in the amount of ctDNA are associated with a tumor response to therapies in metastatic cancer, including anti-EGFR and check point inhibitor therapy in lung cancer [21,22], Palbociclib and Fulvestrant [23], anti-HER2 therapy [24] and AKT inhibitor with Paclitaxel [25] in metastatic breast cancer. O’Leary et al. [23] defined a circulating tumor DNA ratio (CDR) by dividing the quantity of ctDNA at an on-treatment time point by the baseline quantity of the same ctDNA. All of the aforementioned studies were performed using specific assays targeting specific genes and gene variants that are commonly observed in the selected cancers under study. Our approach was to use a tumor agnostic approach to measure tumor DNA in the plasma, based on the inherent genomic instability of tumor DNA.

Using a low-coverage, genome-wide sequencing approach, with a predetermined cut-off point determined by the analysis of over 6000 normal samples [16], we were able to detect genomically unstable DNA, as determined by the presence of CNAs in cfDNA from the plasma of 64% of patients with metastatic breast cancers. Using matched cfDNA and tumor tissue, it was determined that approximately a 1% to 2% tumor fraction is necessary to reach the detection threshold of 170. It is possible that, in patients without a detectable GIN, not enough cfDNA was available to detect the copy number aberrations or that the amplitude of tumor-specific CNAs was insufficient to elevate the GIN above the established threshold. Interestingly, the one patient that underwent surgery and radiation before treatment initiation (no measurable metastatic disease) had no evidence of disease throughout the clinical follow-up that lasted more than five years, which can explain the lack of a detectable GIN in all samples analyzed from this patient. 

In order to associate early on-treatment changes in ctDNA with the response to the therapy and outcome, we analyzed samples collected approximately a week and two weeks after treatment initiation. We found that GIN values above the predetermined threshold at these two time points and at the baseline had a prognostic value with an overall survival at least doubling when an elevated GIN was not observed. The sample size was too small to allow significant correlations between the clinical and tumor features and baseline GIN detection, although we did find that patients with TNBC had significantly higher GIN values than patients with non-TNBC tumors, consistent with the high genomic instability in TNBCs [26,27].

As with the more targeted sequencing studies, we also noted a rapid decrease in the ctDNA values early during treatment. The GIN values at one week and at about two weeks after treatment initiation were associated with an early clinical response (at three months) but not with the clinical response at six months. Moreover, the average decrease in GIN from baseline to T2 and from T1 to T2 was associated with the three-month response, while a GIN decrease from baseline to T1 above the median was associated with improved overall survival. Interestingly, we observed that declines (≥50%) of the baseline GIN value at one week allowed to better predict the response at three months (PPV = 100%) than decreases during the second week (PPV = 89%). These encouraging results with a small patient cohort provide an initial proof-of-concept but warrant further validation of this test in a larger patient cohort. 

Two patterns of dynamic changes in GIN levels early during treatment were observed. One pattern showed a continuous decrease in GIN during the first two weeks of treatment and had a good predictive value for the response at three months (88%) but not at six months (43%). The second pattern showed a decreased GIN during the first week, followed by an increase in GIN during week two, and had an NPV for a nonresponse (or progressive disease) of 80% at six months and of 60% at three months. In the metastatic setting, monitoring patients with ctDNA and identifying patients who progress during treatment earlier than with imaging techniques could provide the opportunity to prompt clinical intervention to modify the patient’s line of treatment [28]. However, prospective clinical trials are still needed to determine if early treatment modifications based on ctDNA changes can improve the clinical outcome [29,30]. Interestingly, of five patients presenting the pattern of nonresponse (#2), two presented very aggressive diseases and died within one month of treatment, and for two other patients, additional blood samples were analyzed, and we observed a continuous increase in GIN over time until the time of progression and patient’s death (Figure S9). These interesting preliminary results provide a foundation for a longitudinal prospective study to better understand how early increases in GIN levels can antedate clinical disease progression in metastatic breast cancer patients. 

## 5. Conclusions

Our findings are consistent with the value of early testing of ctDNA (e.g., one to two weeks) upon the initiation of a novel treatment in metastatic breast cancer as a predictor of the early treatment response (three months) and overall survival. Our study highlights the values of early on-treatment ctDNA measurements and provides an approach that does not need to rely on tumor sequencing or specific panels, thus making it much more clinically feasible.

## Data Availability

The data presented in this study are available on request from the corresponding author. The data are not publicly available due to privacy considerations.

## Supplementary Materials

The following are available online at https://www.mdpi.com/2072-6694/13/6/1331/s1: Figure S1: CONSORT flow diagram for the entire cohort of patients enrolled in this study, Figure S2: Percentage of tumor DNA required for the detection of GIN values above the predefined threshold, Figure S3: Undetectable GIN values in metastatic patients with no measurable disease, Figure S4: Correlation of baseline GIN levels with the number of lines of treatment and number of metastatic sites, Figure S5: Progression free survival (PFS) and GIN detection and changes at different time points, Figure S6: Patterns of dynamic changes in GIN values related to the response at 3 months, Figure S7: Patterns of dynamic changes in GIN values related to the response at 6 months, Figure S8: GIN levels at the baseline and changes at the T1 and T2 time points for the 3 BC subtypes, Figure S9: GIN values in 2 patients with a rise of the T2 dynamic pattern with long-term follow-up, Table S1: GIN levels and clinical data.
