# Supplementary material for: Early, On-Treatment Levels and Dynamic Changes of Genomic Instability in Circulating Tumor DNA Predict Response to Treatment and Outcome in Metastatic Breast Cancer Patients
Source: Cancers (Basel). 2021 Mar 16;13(6):1331. doi: 10.3390/cancers13061331 (PMC7999382; doi:10.3390/cancers13061331)
Supplement: Supplementary file 1 [file cancers-13-01331-s001.pdf]

Early, On-Treatment Levels and Dynamic Changes of Genomic Instability in Circulating Tumor DNA Predict Response to Treatment and Outcome in Metastatic Breast Cancer Patients.

Adriana Aguilar-Mahecha ,Josiane Lafleur ,Susie Brousse ,Olga Savichtcheva ,Kimberly A. Holden,Nathan Faulkner,Graham McLennan,Taylor J. Jensen and Mark Basik

Supplementary data

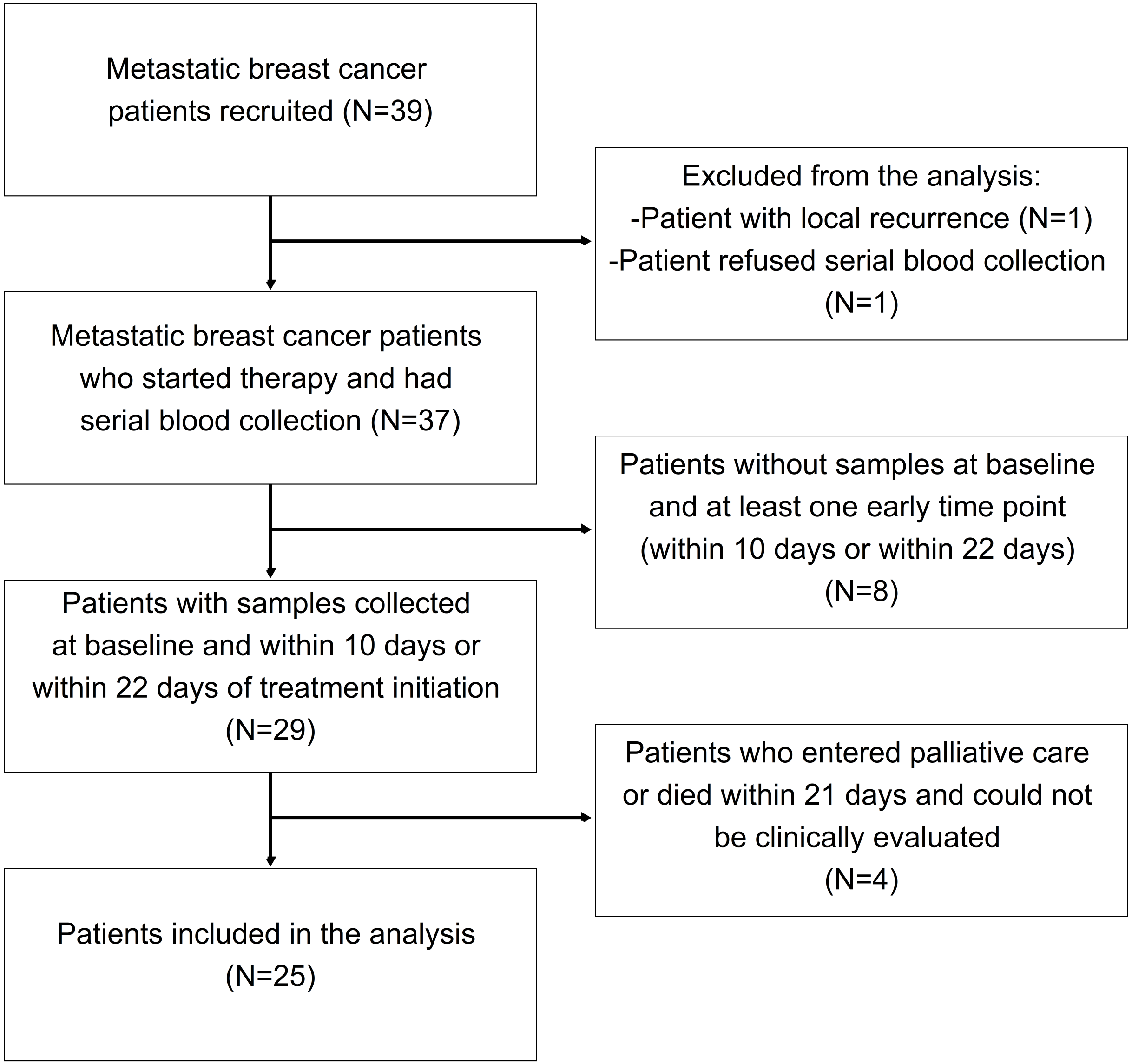

Figure S1. CONSORT flow diagram for the entire cohort of patients enrolled in this study.

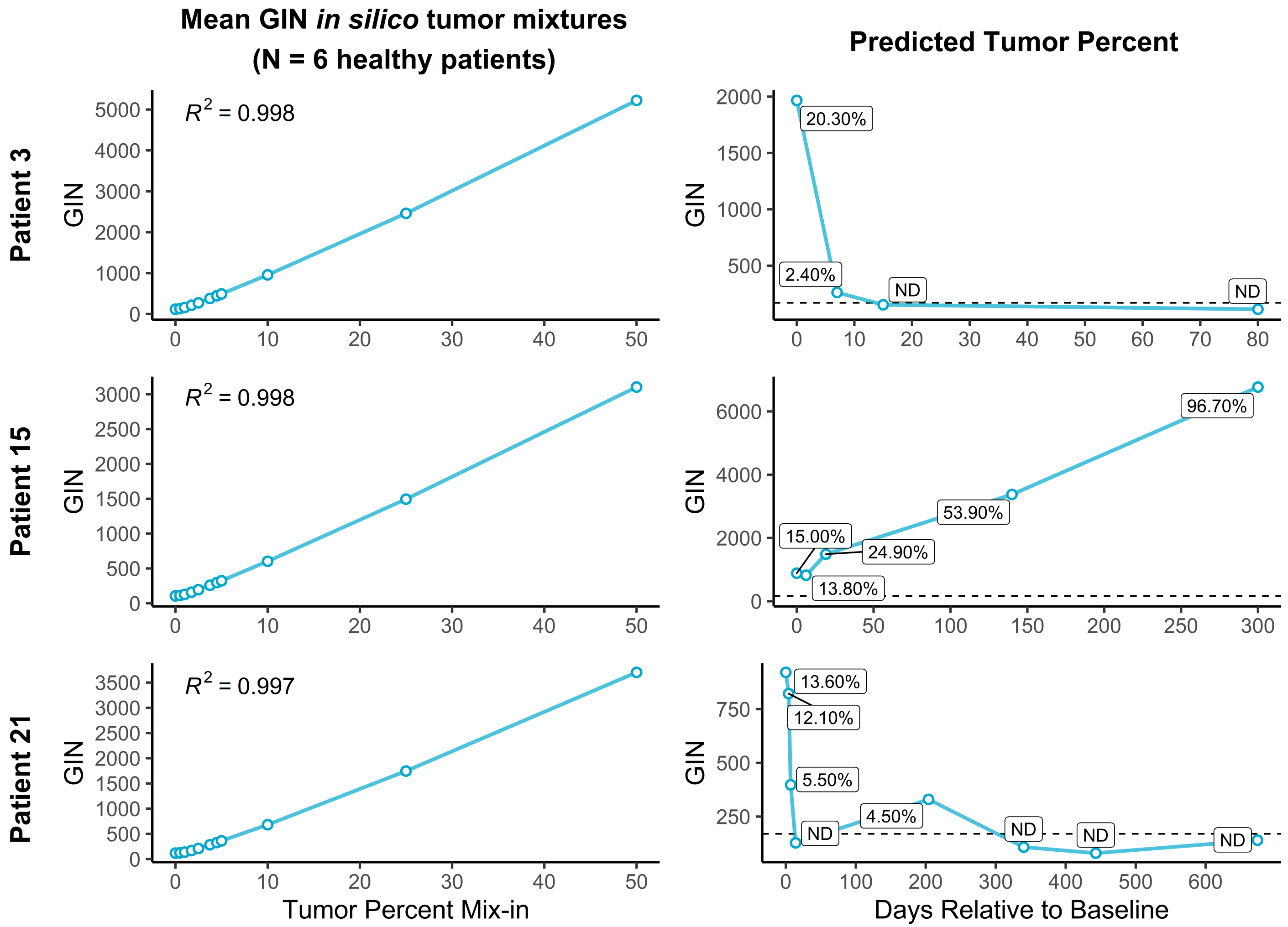

Figure S2. Percentage of tumor DNA required for the detection of GIN values above the pre-defined threshold. On the left, the DNA extracted from the plasma of 3 patients with metastatic breast cancer (Patients 3, 15 and 21) is diluted with DNA from the plasma of a pool of 6 healthy patients. Loess Regression analysis is shown with the X-axis as the tumor percent mix-in and y-axis with the GIN value. As little as 1-2 % of tumor DNA is sufficient to reach a detectable (>170) GIN value in cfDNA. On the right, the serial GIN value curves are annotated with estimated % tumor DNA in the plasma sample in blood samples from the same 3 patients.

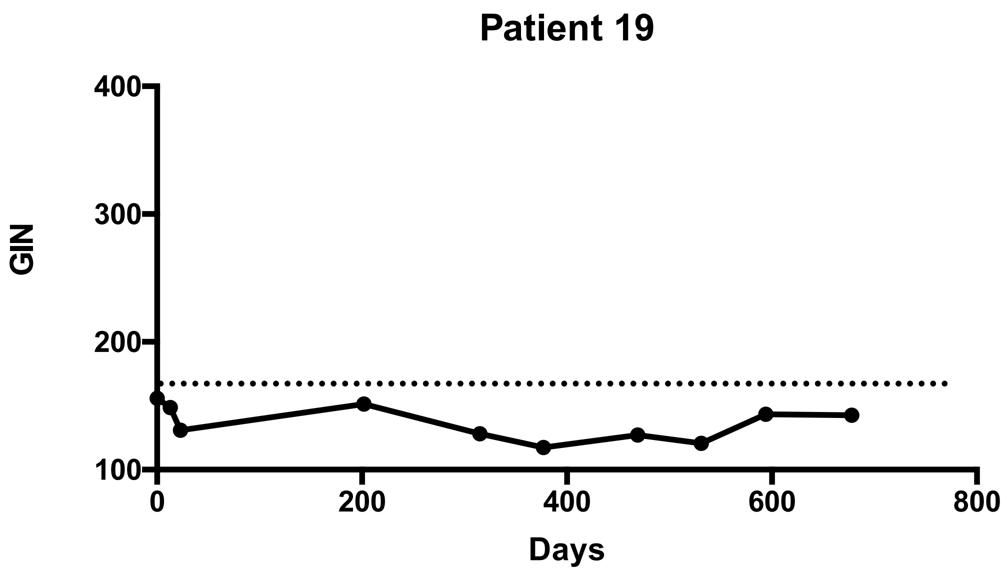

Figure S3. Undetectable GIN values in metastatic patient with no measurable disease (patient 19).
The patient had undergone surgery for brain metastasis and radiotherapy for bone metastasis before treatment, and at baseline, there was no active metastatic disease. During follow-up disease did not recur and GIN values remained below threshold for over 2 years of follow-up.

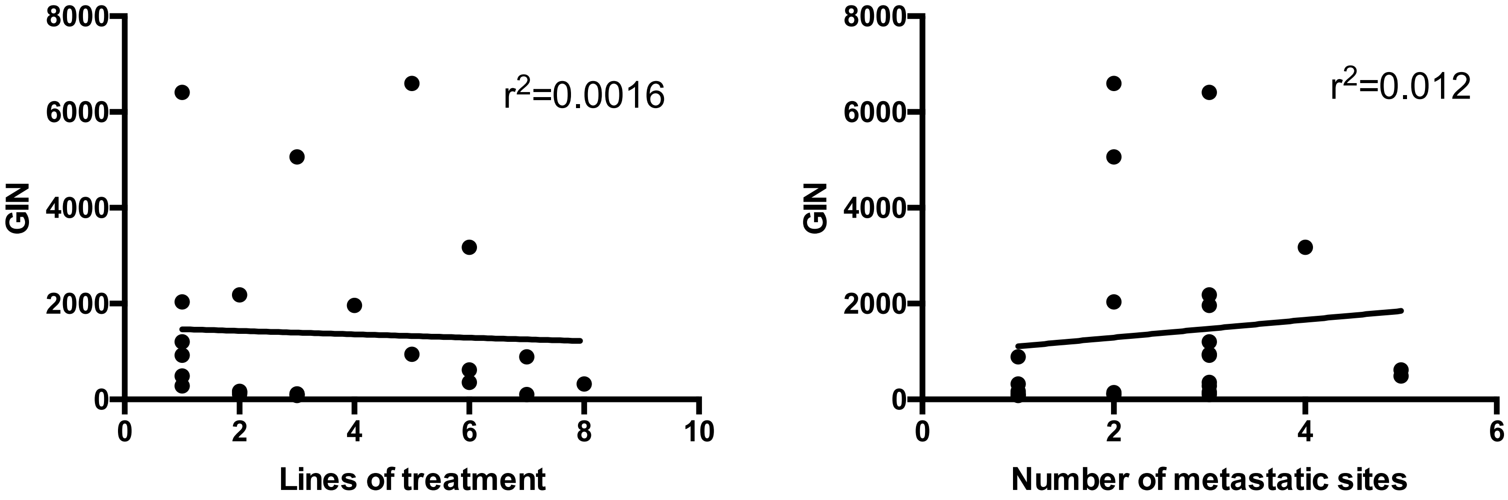

Figure S4. Correlation of baseline GIN values with number of lines of treatment (left) and number of metastatic sites at the time of treatment initiation (right). There appears to be no correlation of baseline GIN with either factor.

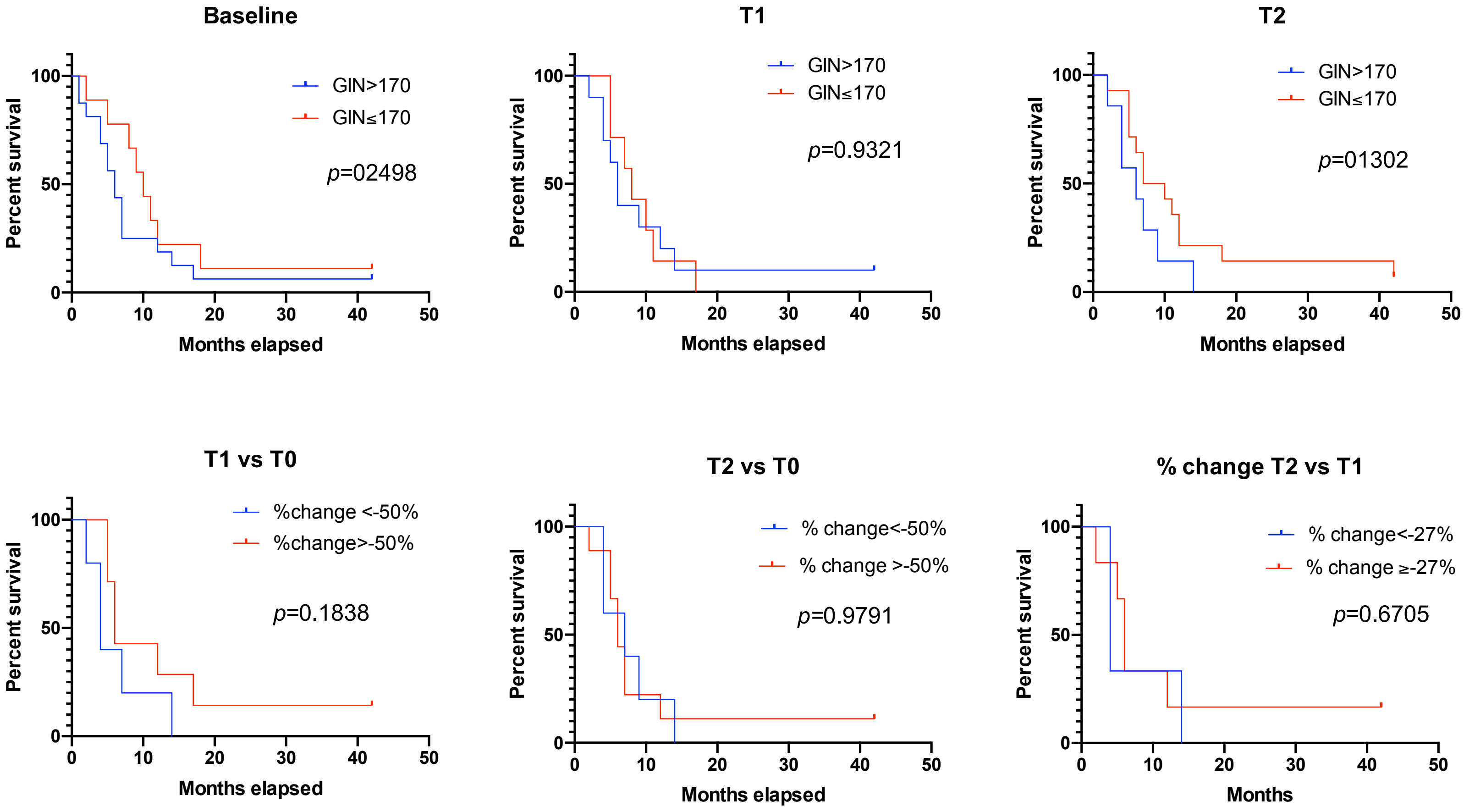

Figure S5. Progression free survival (PFS) and GIN detection and changes at different time points. Top: PFS of patients with plasma GIN values above and below pre-established threshold (170) at 3 different time points. Baseline (left); T1 (average 1 week post-treatment) (middle); T2 (average 2 weeks post-treatment) (right).Bottom: PFS of patients with plasma % change in GIN values above and below pre-established threshold (-50%) or the median (-27%). Left: % change T1 vs T0; middle: T2 vs T0; right: T2 vs T1.

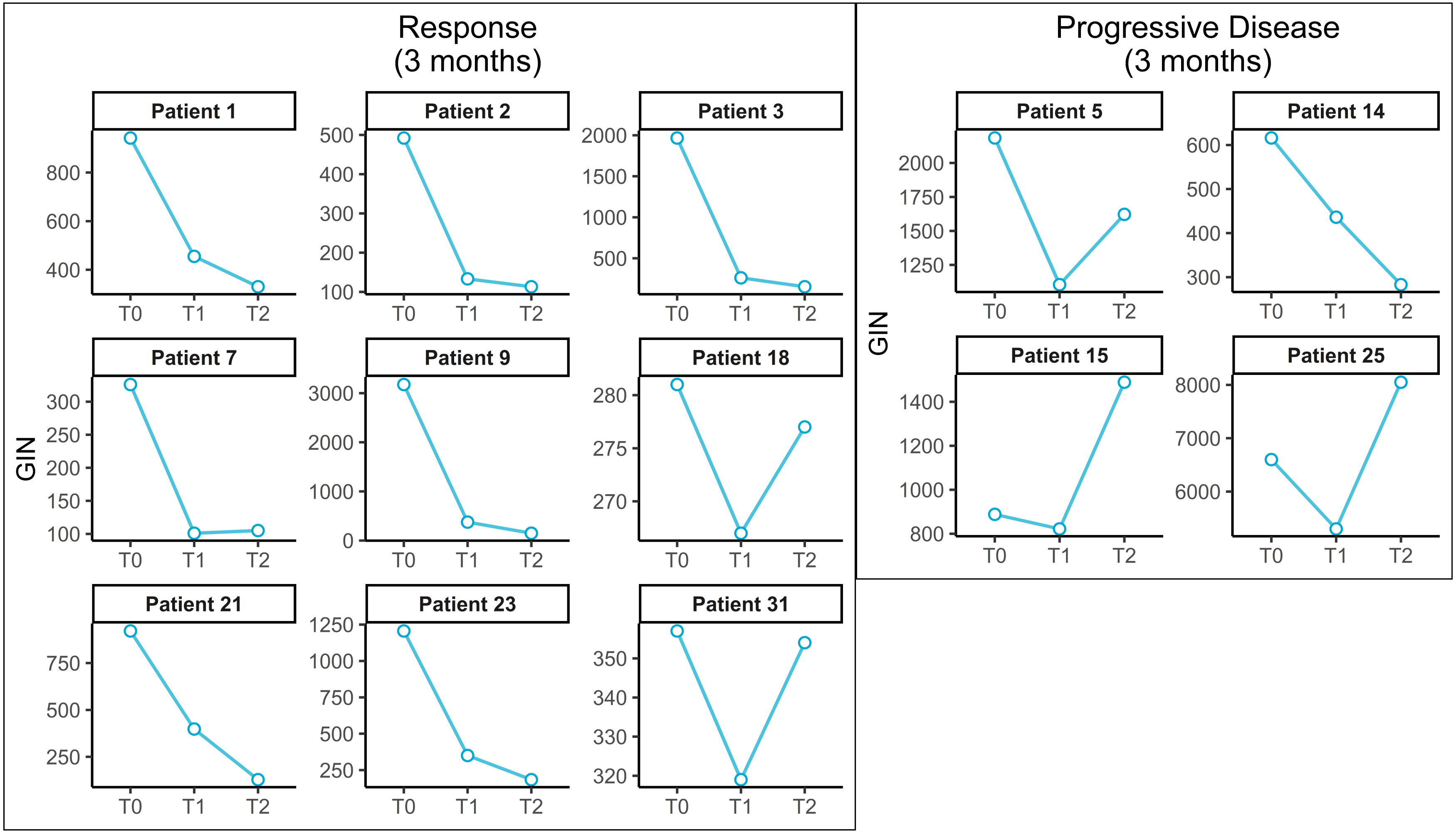

Figure S6. Patterns of dynamic changes in GIN values related to response at 3 months. The patterns of rise and fall of GIN values across the 3 week period after treatment initiation is displayed here. On the left are responding patients (partial response and stable disease) and the right, patient with progressive disease (PD) evaluated at 3 months post-treatment initiation. Note that in 3 of the 4 patients with PD, the GIN values rise from T1 to T2, whereas this is observed in only 2 of the 9 responding patients. Only 1 of the 4 patients with PD does not show a rise at T2, a pattern observed in 7 of the 9 responding patients.

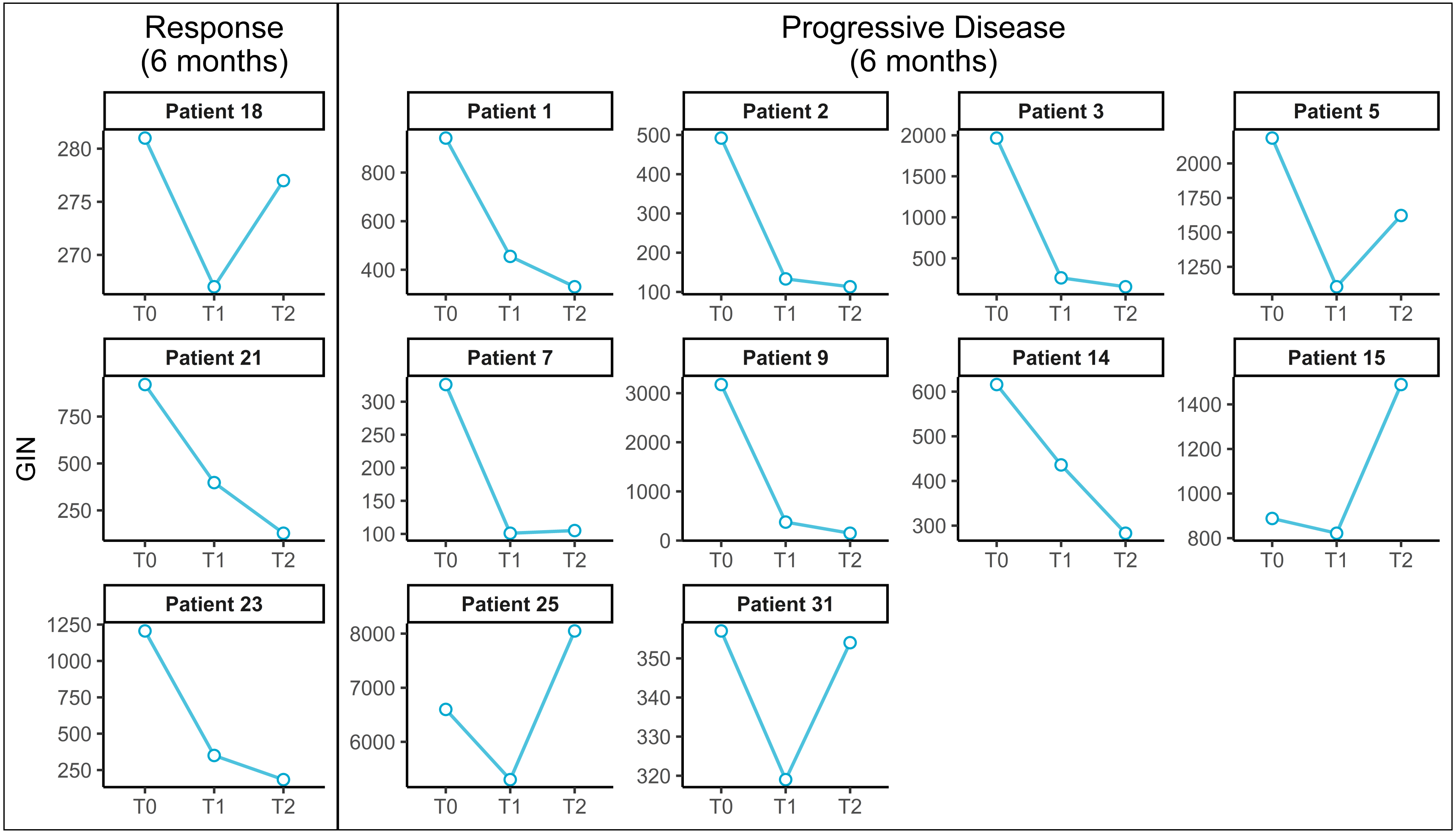

Figure S7. Patterns of dynamic changes in GIN values related to response at 6 months. The patterns of rise and fall of GIN values across the 3 week period (T0, T1, T2) after treatment initiation is displayed here. On the left are responding patients (partial response and stable disease) and the right, patient with progressive disease (PD) evaluated at 6 months post-treatment initiation. Note that in 4 of the 10 patients with PD, the GIN values rise from T1 to T2, whereas this is observed in only 1 of the 3 responding patients.

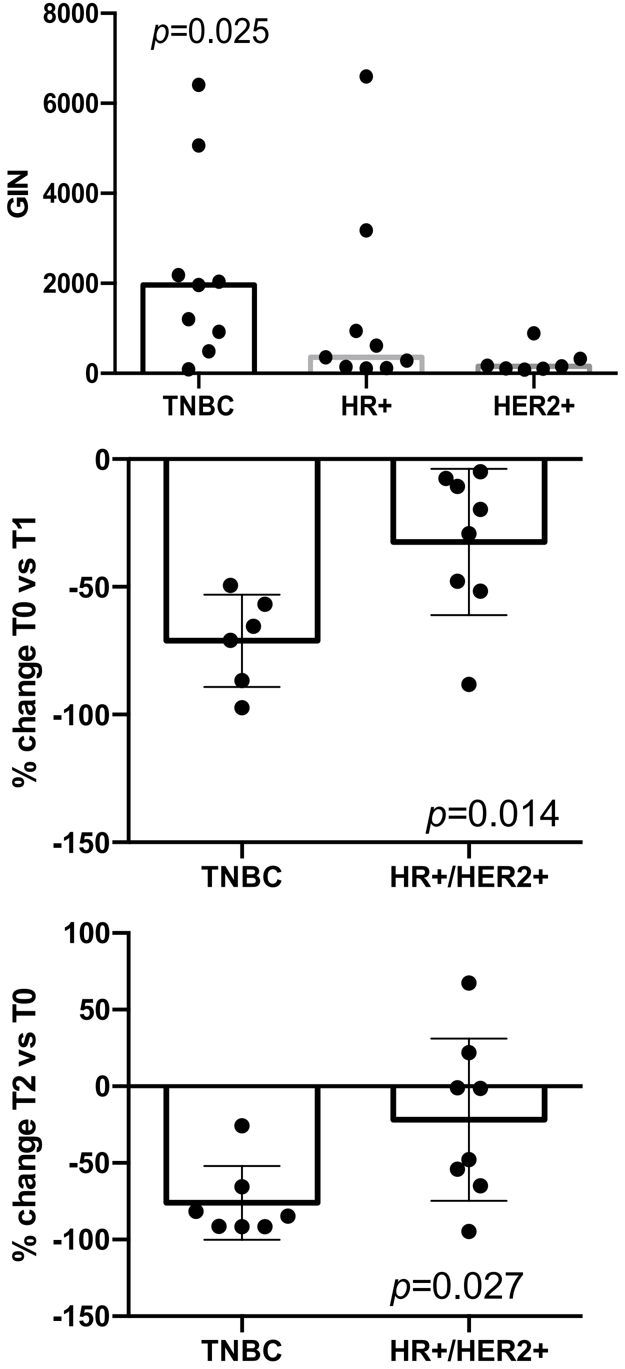

Figure S8. GIN levels at baseline and changes at T1 and T2 time points for the 3 BC subtypes. Bar graphs and scatter plots showing GIN values at baseline (top), % change in GIN from T0 to T1 (middle) and % change in GIN from T2 to T0 (bottom). Error bars represent standard errors of the mean. TNBC: triple negative breast cancer; HR: hormone receptor; HER2: Human Epidermal growth factor Receptor 2. P values calculated using ANOVA.

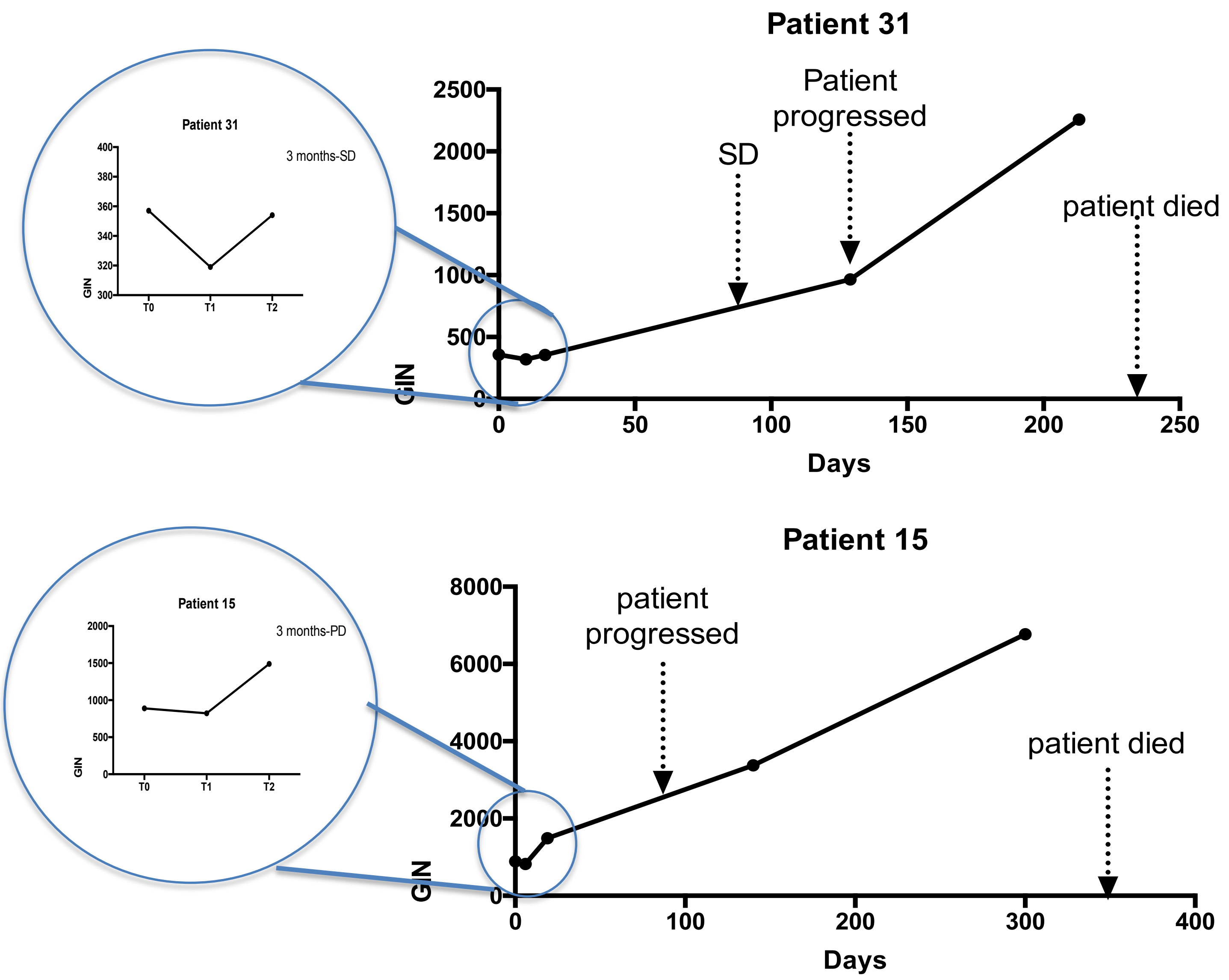

Figure S9. GIN values in 2 patients with rise at T2 dynamic pattern with longer term follow-up. 2 of 5 patients with GIN rise at T2 relative to T1 showed a continuous increase in GIN over time until the time of progression and patient's death. SD : Stable disease; PD: progressive disease. Inset shows magnified GIN values and response at 3 months.

Table S1. Clinical characteristics

Pt N°	Sub-type	Metastatic sites	N°	Treatment	Line	T0 GIN	T1 (days)	GIN T1	T1/T0 (%)	T2 (days)	GIN T2	T2/T0 (%)	T2/T1 (%)	R 3mths	R 6mths	PFS (mths)	OS (mths)
1	HR+	Bone, liver, lung	3	Paclitaxel	5	942	7	455	-52	14	330	-65	-27	SD	PD	6	16
2	TNBC	LN, bone, lung, brain, nervous	5	Carbo/Taxol	1	492	7	133	-73*	14	113	-65*	ND	PR	PD	5	30
3	TNBC	Skin, LN, lung	3	Carbo/Gem	4	1966	7	262	-87	15	153	-91*	-35	PR	PD	5	9
4	HR+	Bone	1	Capecitabine	3	115	8	240	NA	14	448	NA	NA	SD	SD	9	12
5	TNBC	Skin, LN, bone	3	Vinorelbine	2	2184	6	1105	-49	19	1622	-26	47	PD[#]	PD[#]	0	1
7	Her2+	Lung	1	Eribulin	8	326	6	101	-48*	13	105	-48*	ND	SD	PD	6	15
9	HR+	LN, bone, liver, lung	4	Eribulin	6	3176	6	376	-88	20	149	-95*	-55	PR	PD	6	26
11	TNBC	Bone, lung	2	PacliT	3	5062	NA	NA	NA	14	428	-92	NA	PR	PD	6	24
12	TNBC	Skin	1	Caelyx	3	92	7	145	ND	14	110	ND	ND	PR	PD	5	46[δ]
14	HR+	Skin, LN, soft tissue, bone, brain	5	PacliT	6	616	6	436	-29	13	283	-54	-35	PD	PD[#]	2	5
15	Her2+	Liver	1	Herceptin/Carbo	7	888	6	822	-8	19	1489	168	81	PD	PD	3	12
16	Her2+	Skin	1	PacliT/Herceptin/Perjeta	2	167	8	111	ND	15	104	ND	ND	PR	CR	11	33
17	HR+	LN, lung	2	Carbo/Taxol	2	142	6	78	ND	13	120	ND	ND	PR	CR	10	41
18	HR+	Bone, liver, lung	3	PacliT/Letrozole	1	281	5	267	-5	14	277	-1	4	PR	SD	14	30
19	Her2+	Bone, brain, ovary	3	DoceT/Herceptin/Perjeta	2	156	NA	NA	NA	13	149	ND	NA	CR	CR	42[Ψ]	42[δ]
20	Her2+	Lung, brain, nervous spine	3	Carbo/Herceptin	7	103	NA	NA	NA	11	98	ND	NA	PD	PD	2	14
21	TNBC	LN, chest wall, lung	3	Carbo/T	1	921	7	398	-57	14	128	-82*	-57	CR	CR	42[Ψ]	42[δ]
22	Her2+	Lung, brain	2	Carbo/Herceptin	3	108	9	129	ND	NA	NA	NA	NA	SD	SD	8	42[δ]
23	TNBC	Bone, liver, omentum	3	Carbo/Cycloph.	1	1206	7	350	-71	21	184	-85	-47	SD	PR	12	21
25	HR+	Bone, liver	2	Vinorelbine	5	6599	6	5300	-20	13	8050	22	52	PD[#]	PD[#]	0	1
28	TNBC	LN, bone, lung	3	Abraxane/Atezolizumab	1	6413	7	107	-97*	NA	NA	NA	NA	PR	PR	17	30
29	TNBC	Lung, brain	2	Abraxane/Atezolizumab	1	2035	NA	NA	NA	14	129	-92*	NA	SD	SD	7	20
31	HR+	Liver, lung, omentum	3	Doxorubicin	6	357	6	319	-11	13	354	-1	11	SD	PD	4	8
34	Her2+	Lung	1	T-DM1/Atezolizumab	3	80	NA	NA	NA	21	94	ND	NA	PR	PR	18	39[δ]
37	HR+	Bone, Liver	2	Letrozole/Ribociclib	2	112	NA	NA	NA	14	148	ND	NA	SD	SD	12	28
		Average	2		3	1382	7	586	-49	15	655	-47	-6			11	23
		SD	1		2	1942	1	1171	31	3	1662	49	48			11	14
		Median	3		3	492	7	267	-51	14	149	-65	-27			7	24
		Min	1		1	80	5	78	-97	11	94	-95	-57			0	1
		Max	5		8	6599	9	5300	-5	21	8050	67	81			42	46

Non-detectable GIN, *difference calculated with 170 for ND values, [#]patients who were dead at 3 or 6 months, [Ψ]patients with no evidence of disease, [δ]patients alive.
